# Amniotic fluid stem cells ameliorate cisplatin-induced acute renal failure through induction of autophagy and inhibition of apoptosis

**DOI:** 10.1186/s13287-019-1476-6

**Published:** 2019-12-04

**Authors:** Ekta Minocha, Rohit Anthony Sinha, Manali Jain, Chandra Prakash Chaturvedi, Soniya Nityanand

**Affiliations:** 1 Stem Cell Research Centre, Department of Hematology, Sanjay Gandhi Post Graduate Institute of Medical Sciences, Rae Bareli Road, Lucknow, UP 226014 India; 2 Department of Endocrinology, Sanjay Gandhi Post Graduate Institute of Medical Sciences, Lucknow, India

**Keywords:** Amniotic fluid stem cells, Cisplatin, Acute renal failure, Apoptosis, Autophagy, Chloroquine

## Abstract

**Background:**

We have recently demonstrated that amniotic fluid stem cells (AFSC) express renal progenitor markers and can be differentiated in vitro into renal lineage cell types, viz, juxtaglomerular and renal proximal tubular epithelial-like cells. Here, we have evaluated the therapeutic efficacy of AFSC in a cisplatin-induced rat model of acute renal failure (ARF) and investigated the underlying mechanisms responsible for their renoprotective effects.

**Methods:**

ARF was induced in Wistar rats by intra-peritoneal injection of cisplatin (7 mg/kg). Five days after cisplatin injection, rats were randomized into two groups and injected with either AFSC or normal saline intravenously. On days 8 and 12 after cisplatin injection, the blood biochemical parameters, histopathological changes, apoptosis and expression of pro-apoptotic, anti-apoptotic, and autophagy-related proteins in renal tissues were studied in both groups of rats. To further confirm whether the protective effects of AFSC on cisplatin-induced apoptosis were dependent on autophagy, chloroquine, an autophagy inhibitor, was administered by the intra-peritoneal route.

**Results:**

Administration of AFSC in ARF rats resulted in improvement of renal function and attenuation of renal damage as reflected by significant decrease in blood urea nitrogen, serum creatinine levels, tubular cell apoptosis as assessed by Bax/Bcl2 ratio, and expression of the pro-apoptotic proteins, viz, PUMA, Bax, cleaved caspase-3, and cleaved caspase-9, as compared to the saline-treated group. Furthermore, in the AFSC-treated group as compared to the saline-treated group, there was a significant increase in the activation of autophagy as evident by increased expression of LC3-II, ATG5, ATG7, Beclin1, and phospho-AMPK levels with a concomitant decrease in phospho-p70S6K and p62 expression levels. Chloroquine administration led to significant reduction in the anti-apoptotic effects of the AFSC therapy and further deterioration in the renal structure and function caused by cisplatin.

**Conclusion:**

AFSC led to amelioration of cisplatin-induced ARF which was mediated by inhibition of apoptosis and activation of autophagy. The protective effects of AFSC were blunted by chloroquine, an inhibitor of autophagy, highlighting that activation of autophagy is an important mechanism of action for the protective role of AFSC in cisplatin-induced renal injury.

## Background

Acute renal failure (ARF) also known as acute kidney injury (AKI) is a grave clinical condition characterized by sudden loss of renal function and can be induced by a variety of factors including hypoxia, drugs, mechanical trauma, inflammation, surgery, cardiopulmonary bypass, and hemodynamic instability [1]. In some cases ARF recovers spontaneously, while in others the recovery process is either delayed or does not occur at all, thus leading to chronic kidney disease (CKD). Therefore, it becomes very necessary to restore the normal structure and function of the kidney after ARF in order to prevent its progression into CKD.

Over the last decade, stem cell-based therapy has emerged as a promising approach for renal regeneration, and of the various stem cells used, mesenchymal stromal cells (MSCs) have reached phase I/II clinical trials. However, even with MSC therapy, the results are conflicting, and no consistent benefit has been demonstrated till date, perhaps due to their limited differentiation potential in vivo [2]. Therefore, the search to identify a suitable stem cell type for renal regeneration is still on.

Amniotic fluid stem cells (AFSC) represent a novel class of stem cells with intermediate characteristics between embryonic stem cells and adult stem cells. They have extended self-renewal capacity and multipotent differentiation potential [3]. Major volume of amniotic fluid is derived from fetal urine [4], and we have previously shown that it harbors a stem cell population that expresses high percentage of renal progenitor markers and possesses renal differentiation potential [5, 6]. Although previous studies have shown the renal regenerative potential of AFSC in cisplatin- or glycerol-induced AKI [7–11], the underlying mechanisms responsible for the renoprotective effect are largely unknown.

It has been shown that autophagy has a protective role in cisplatin-induced AKI by inhibiting apoptosis [12]. Autophagy also plays a critical role in maintaining homeostasis of renal tubular cells [13], and impairment of autophagy in renal tubular cells has been implicated in the pathogenesis of various kidney diseases, including AKI [14, 15]. Thus, restoration and promotion of autophagy is considered as a promising therapeutic strategy in ARF.

Therefore, the aim of the present study was to investigate the renoprotective effects of AFSC in cisplatin-induced rat model of ARF and to evaluate the role of autophagy in AFSC-mediated amelioration of ARF.

## Methods

### Animals and ethics statement

Adult Wistar rats weighing 200–225 g were used in the study. The animals were maintained on a 12-h light-dark cycle in a constant temperature and humidity environment, with food and water provided ad libitum*.* All animal procedures in the study were conducted in accordance with the guidelines of Institutional Animal Ethics Committee (IAEC) and Committee for the Purpose of Control and Supervision of Experiments on Animals (CPCSEA), India. The protocol was approved by IAEC of Sanjay Gandhi Post Graduate Institute of Medical Sciences, Lucknow, India.

### Isolation and culture of amniotic fluid stem cells (AFSC)

Amniotic fluid samples were obtained from pregnant female Wistar rats at gestation day 16 and cultured as previously described [5]. Briefly, from each gravid rat, 2–3 ml of amniotic fluid was obtained, corresponding to cell numbers ranging from 7 × 10^3^ to 7 × 10^5^ which was then centrifuged at 300*g* for 5 min and the pellet obtained was resuspended in complete culture medium consisting of α-MEM, 16.5% fetal bovine serum, 2 mM Glutamax, 100 U/ml penicillin, and 100 μg/ml streptomycin (all from Gibco, NY, USA) and incubated at 37 °C with 5% CO_2_ atmosphere. After 72 h of seeding, culture media containing non-adherent cells were replaced. On day 7, the adherent cells were harvested by trypsinization with TrypLE Express (Gibco, NY, USA) and further expanded as above. The third passage cells were used throughout the study.

### Flow cytometry

Flow cytometry was performed on three independent amniotic fluid samples (*n* = 3) (obtained from three independent gravid rats) to characterize AFSC for the expression of (i) cell-surface mesenchymal markers using CD90, CD73, CD105, hematopoietic marker: CD45, and MHC-Class II and (ii) intracellular renal progenitor markers: Wilms’ tumor protein 1 (WT1), Paired Box 2 (PAX2), and SIX Homeobox2 (SIX2) as previously described [5]. All flow-cytometric acquisitions were performed on BD-FACS CantoII and analyzed using FACS Diva software. The specific dilutions and details of the antibodies used are listed in Table 1.

**Table 1:** List of antibodies with their dilutions, sources, and catalog numbers

Antibody	Dilution	Source
Mouse monoclonal CD90 (Cat# 554894)	1:100 (FCM)	BD Biosciences, CA, USA
Mouse monoclonal CD73 (Cat# 551123)	1:100 (FCM)	BD Biosciences, CA, USA
Rat monoclonal CD105 **(**Cat# sc-71042)	1:100 (FCM)	Santa Cruz Biotechnology, CA, USA
Mouse monoclonal CD45 (Cat# 554878)	1:100 (FCM)	BD Biosciences, CA, USA
Mouse monoclonal MHC Class II (Cat# ab22266)	1:100 (FCM)	Abcam, MA, USA
Mouse monoclonal WT1 (IgG clone #6F-H2)	1:100 (FCM)	My BioSource, CA, USA
Rabbit monoclonal Pax2 (Cat# ab79389)	1:100 (FCM)	Abcam, MA, USA
Goat polyclonal Six2 (Cat# sc-67834)	1:100 (FCM)	Santa Cruz Biotechnology, CA, USA
Rabbit monoclonal Phospho-AMPKα (Cat# 2535)	1:1000 (WB)	Cell Signaling Technology (CST), MA, USA
Mouse monoclonal Phospho-p70 S6 Kinase (Cat# 9206)	1:1000 (WB)	CST, MA, USA
Rabbit monoclonal ATG5 (Cat#12994)	1:1000 (WB)	CST, MA, USA
Rabbit monoclonal ATG7 (Cat# 8558)	1:1000 (WB)	CST, MA, USA
Rabbit monoclonal Beclin1 (Cat# 3495)	1:1000 (WB)	CST, MA, USA
Rabbit polyclonal LC3B (Cat# 2775)	1:1000 (WB)	CST, MA, USA
Mouse monoclonal cleaved-Caspase-9 (Cat# 9508)	1:1000 (WB)	CST, MA, USA
Rabbit polyclonal SQSTM1/p62 (Cat# ab91526)	1:1000 (WB)	Abcam, MA, USA
Rabbit polyclonal SQSTM1/p62 (Cat# PM045)	1:200 (IHC)	MBL International, MA, USA
Rabbit polyclonal p53 (Cat# ab131442)	1:1000 (WB)	Abcam, MA, USA
Rabbit polyclonal Bcl-2 (Cat# ab196495)	1:1000 (WB)	Abcam, MA, USA
Rabbit monoclonal Bax (Cat# ab32503)	1:1000 (WB)	Abcam, MA, USA
Rabbit polyclonal PUMA (Cat# ab9643)	1:1000 (WB)	Abcam, MA, USA
Rabbit polyclonal cleaved- Caspase-3 (Cat# 9661)	1:1000 (WB) 1:100 (IHC)	CST, MA, USA
Rabbit monoclonal GAPDH (Cat# 5174)	1:1000 (WB)	CST, MA, USA
Biotinylated LTL (Cat# B-1325)	1:500 (IHC)	Vector Laboratories, CA, USA
Goat Anti-mouse IgG (H&L)(PE) (Cat#ab97041)	1:200 (FCM)	Abcam, MA, USA
Goat Anti-rabbit IgG (H&L)(FITC) (Cat#ab6717)	1:200 (FCM)	Abcam, MA, USA
Streptavidin, Alexa Fluor 568 conjugate (Cat#S11226)	1:500 (IHC)	Thermo Fisher Scientific, MA, USA
Goat Anti-Rabbit (Alexa Flour 488) (Cat# ab150077)	1:500 (IHC)	Abcam, MA, USA
Goat Anti-Rabbit (HRP) (Cat# ab205718)	1:5000 (WB)	Abcam, MA, USA
Goat Anti-mouse (HRP) (Cat# ab205719)	1:5000 (WB)	Abcam, MA, USA

### Development of ARF model and AFSC therapy

ARF was induced in male Wistar rats weighing 200–225 g by an intra-peritoneal injection of cisplatin (Sigma Aldrich, MO, USA) at a dose of 7 mg/kg of body weight after fasting for 12 h. After 5 days of cisplatin injection, a significant increase in the blood biochemical parameters and renal damage was observed; hence, at this point, the rats were randomized into two groups: AFSC-treated group (*n* = 10) and saline-treated group (*n* = 10) for the evaluation of efficacy of stem cell therapy. On day 5 after cisplatin injection, AFSC (2 × 10^6^cells/rat) suspended in 500 μl of normal saline (in AFSC-treated group) or 500 μl saline alone (saline-treated group) was injected intravenously through tail vein in each rat (Additional file 1: Figure S1A). In addition, a group of healthy control rats (*n* = 5) was also included in the study to compare the histology and renal function with the AFSC- and saline-treated groups.

To determine the homing ability of AFSC to cisplatin-injured kidney, the AFSC were first transduced with CellLight Nucleus-GFP, BacMam 2.0 (Thermo Fisher Scientific, MA, USA) at a concentration of 45 PPC (particles per cell) according to the manufacturer’s instruction and then administered intravenously in cisplatin-induced ARF rats (*n* = 3) at a concentration of 2 × 10^6^ cells/rat suspended in 500 μl of normal saline. After 3 days, the rats were sacrificed and kidney tissues were obtained that were embedded in paraffin, sliced into 5-μm-thick sections, and then incubated with *Lotus tetragonolobus* Lectin (LTL) for 2 h at room temperature followed by incubation with Streptavidin Alexa Fluor 568 conjugated secondary antibody for 1 h at room temperature. Nuclei were stained with Hoechst dye. Images were acquired using a fluorescence microscope (Olympus BX61) equipped with Nuance Multispectral Imaging System (CRi Inc., MA, USA). GFP-positive cells were counted in five renal sections per rat (*n* = 3), and data was expressed as the number of GFP+ cells per 10^5^ renal cells.

To confirm the protective effects of autophagy following cisplatin-induced AKI, chloroquine, an autophagy inhibitor, was used. Chloroquine diphosphate salt (Sigma Aldrich, MO, USA) was administered to rats (*n* = 10) intra-peritoneally at a dose of 60 mg/kg in distilled water 1 day prior to therapy and then daily till sacrifice (Additional file 1: Figure S1B).

Animals were euthanized by CO_2_ overdose after 5 days (day 5), 8 days (day 8), and 12 days (day 12) of cisplatin injection. Both the kidneys were excised, and blood samples were collected to perform the histological analysis and determination of the blood biochemical parameters respectively.

### Determination of blood urea nitrogen (BUN) and creatinine levels

Renal function was assessed by measuring the BUN and creatinine levels. After blood collection, serum levels of BUN and creatinine were measured using biochemical analyzer Selectra Pro M (ELITech, France) and commercially available assay kits from ELITech diagnostic (France) according to the standard protocols provided by the manufacturers.

### Histopathological analysis

The cortical kidney tissues obtained from the left kidney of at least three independent rats from each group were fixed in 10% formalin, embedded in paraffin, cut into 5-μm-thick sections, and then mounted on slides. Sections were then deparaffinized, rehydrated, and stained with hematoxylin and eosin (H&E) to evaluate the histopathological changes in saline-, AFSC-, and chloroquine-treated kidney tissue sections. The H&E stained sections were then analyzed under a light microscope (OlympusBX51) equipped with a digital camera. Quantitative assessment of renal tubular necrosis was done using the Jablonski grading scores [16].

### Western blotting

The cortical kidney tissues obtained from the right kidney of at least three independent rats from each group, viz, healthy control, saline-treated, AFSC-treated, and chloroquine-treated rats, were homogenized in RIPA Lysis buffer supplemented with 1X protease (cOmplete, EDTA-free protease inhibitor cocktail, Roche) and phosphatase inhibitor cocktail (PhosSTOP, Roche). Kidney tissue homogenate was then incubated on ice for 30 min and then centrifuged at 6000 rpm for 15 min at 4 °C. The supernatant was removed and stored at − 80 °C. Thirty-microgram homogenate protein was loaded and separated by sodium dodecyl sulphate-polyacrylamide gel electrophoresis (SDS-PAGE). After electrophoresis, the proteins were transferred to nitrocellulose membrane. The membranes were blocked with 5% non-fat milk for 1 h at room temperature and incubated with primary antibodies (Table 1), viz phospho-AMP-activated protein kinase (phospho-AMPK), phospho-p70 S6 Kinase (phospho-p70S6K), Autophagy related 5 (ATG5), Autophagy related 7 (ATG7), Beclin1, Microtubule-associated protein 1A/1B-light chain 3 (LC3B), cleaved caspase-9, Sequestosome 1 (SQSTM1/p62), p53, B cell lymphoma 2 (Bcl-2), B cell lymphoma 2-associated X protein (Bax), p53 upregulated modulator of Apoptosis (PUMA), and cleaved caspase-3 at 4 °C overnight. GAPDH was used as loading control. Primary antibodies were detected by corresponding horseradish peroxidase (HRP)-conjugated secondary antibodies using Clarity Western ECL Substrate (Bio-Rad, CA, USA). Semi-quantitative densitometric analysis was performed using ImageJ software.

### Immunofluorescence

The paraffin-embedded kidney tissue sections were deparaffinized, rehydrated, and subjected to antigen retrieval in sodium citrate buffer (10 mM sodium citrate, pH 6.0) at 95 °C for 30 min and then kept at room temperature for cooling. The sections were then blocked in 5% goat serum containing 1% bovine serum albumin (BSA) for 1 h at room temperature followed by primary antibody incubation, viz, p62 and cleaved-caspase3, for 2 h at room temperature in a humidified chamber. After rinsing the slides with PBS, sections were then incubated with Alexa Fluor 488 conjugated secondary antibody for 1 h at room temperature followed by nuclear staining with Hoechst. Images were acquired using a fluorescence microscope (Olympus BX61) equipped with Nuance Multispectral Imaging System (CRi Inc., MA, USA). Sections stained without primary antibody served as negative controls.

### Terminal deoxynucleotidyl-transferase-mediated dUTP nick end labeling (TUNEL) assay

Apoptotic scores in kidney tissue sections were measured by TUNEL assay using an in situ cell death detection kit (Roche, Mannheim, Germany) according to the manufacturer’s instruction. Briefly, the kidney tissue sections were deparaffinized, rehydrated, and subjected to antigen retrieval in citrate buffer followed by labeling with TUNEL reaction mixture for 1 h at 37 °C. LTL was used to identify the proximal tubules. Immunostaining with LTL was performed after TUNEL staining. Sections were then washed in PBS and blocked for 1 h at room temperature in 5% goat serum and 1% BSA, followed by primary antibody incubation, viz, LTL, for 2 h at room temperature in a humidified chamber. After rinsing the slides with PBS, sections were then incubated with Streptavidin Alexa Fluor 568 conjugated secondary antibody for 1 h at room temperature followed by counterstaining with Hoechst dye (Sigma Aldrich, MO, USA). TUNEL-positive nuclei were then counted in 10 randomly selected non-overlapping high power fields (HPF) (40×) in the LTL-positive cortical regions under Nuance Multispectral Imaging System (CRi Inc., MA, USA), and the apoptotic index was expressed as average number of TUNEL^+^ cells/HPF.

### Statistical analysis

Values were expressed as mean ± standard error (SE). One-way analysis of variance (ANOVA) with Bonferroni multiple-comparison post hoc test was used to compare statistically significant differences between groups. Statistical analysis was performed using GraphPad Prism software version 5 (GraphPad, CA, USA), and *p* value < 0.05 was considered statistically significant.

## Results

### Expression of mesenchymal and renal progenitor markers by AFSC

**Fig. 1:**
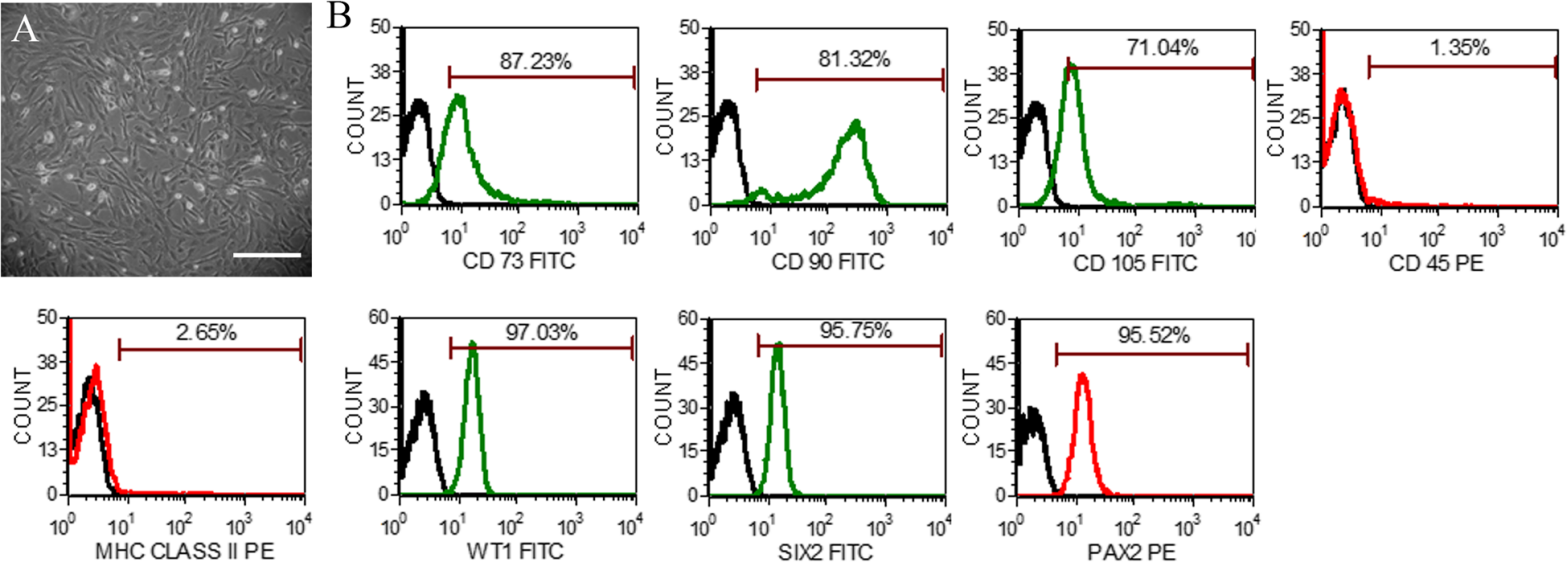
Morphology and phenotypic characterization of AFSC. **a** Representative photomicrographs of amniotic fluid stem cells (AFSC) in culture showing spindle-shaped morphology in passage 3 (scale bar: 100 μm). **b** Phenotypic characterization of AFSC by flow cytometry showing the expression of cell-surface markers, viz, CD73, CD90, CD105, MHC Class II, and CD45, and intracellular renal progenitor markers, viz, WT1, SIX2, and PAX2 (green or red lines detected with FITC- and PE-conjugated antibodies, respectively, and black lines represent isotype controls). PE, phycoerythrin; FITC, fluorescein isothiocyanate

The AFSC exhibited uniform spindle-shaped morphology in culture at passage 3 (Fig. 1a). Flow cytometric analysis showed that AFSC expressed mesenchymal markers, viz, CD73 (87.23% ± 5.16), CD90 (81.32% ± 3.32), and CD105 (71.04% ± 5.09), whereas the expression of CD45 (1.35% ± 1.76) and MHC Class II (2.65% ± 1.51) was found to be less than 5%. Furthermore, AFSC also expressed high percentage of renal progenitor markers, viz, WT1 (97.03% ± 2.24), PAX2 (95.52% ± 3.05), and SIX2 (95.75% ± 3.18), as revealed by flow cytometry (Fig. 1b).

### AFSC therapy promotes improvement of renal function and histology in ARF rats

**Fig. 2:**
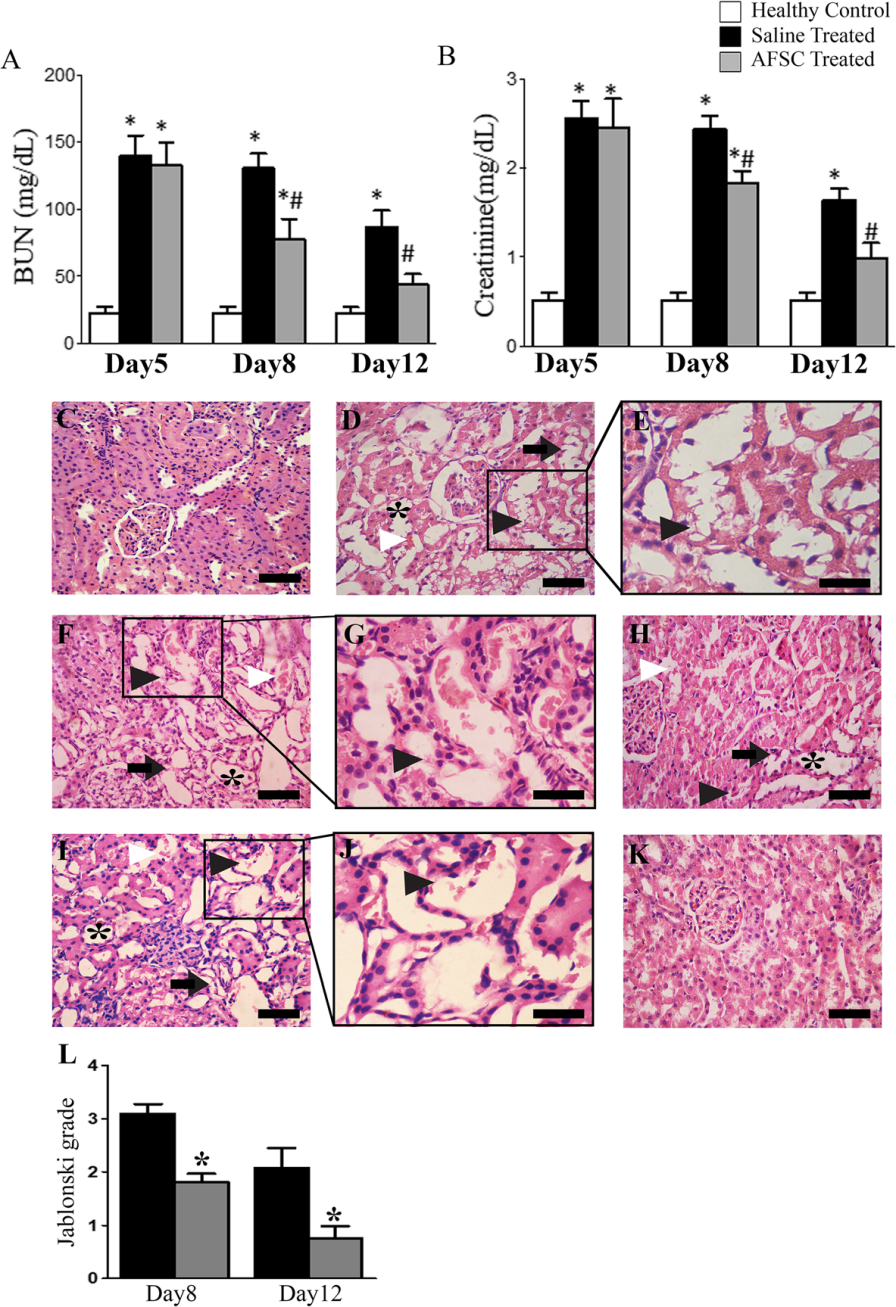
Effect of AFSC therapy on renal function and histology in rats with cisplatin-induced ARF. **a** Levels of blood urea nitrogen (BUN) and **b** serum creatinine measured in healthy controls, saline-treated and AFSC-treated ARF rats on days 5, 8, and 12 after cisplatin injection. Values are expressed as mean ± SE (*p < 0.001 versus healthy control group; ^#^*p* < 0.05 versus saline-treated group). **c** Kidney section of healthy control rat showing normal architecture of tubules and glomeruli (scale bar, 50 μm). **d** Kidney section after 5 days of cisplatin injection showing tubular dilatation (asterisk), necrotic tubules (black arrowhead), intra-tubular cast (white arrowhead), and loss of brush border (black arrow) (scale bar, 50 μm). **e** Magnified image of the boxed area in **d** showing necrotic tubules (black arrowhead) (scale bar, 30 μm). **f** Kidney section of saline-treated ARF rat on day 8 after cisplatin injection showing severe tubular dilatation (asterisk), loss of brush border (black arrow), intra-tubular cast (white arrowhead), and necrotic tubules (black arrowhead) (scale bar, 50 μm). **g** Magnified image of the boxed area in **f** showing necrotic tubules (black arrowhead) (scale bar, 30 μm). **h** Kidney section of AFSC-treated ARF rat on day 8 after cisplatin injection showing signs of recovery as revealed by mild tubular dilatation (asterisk) and fewer necrotic tubules (black arrowhead) and intra-tubular cast (white arrowhead) (scale bar, 50 μm). **i** Kidney section of saline-treated ARF rat on day 12 after cisplatin injection showing few intratubular hyaline casts (white arrowhead), tubular dilatation (asterisk), loss of brush border (black arrow), and necrotic tubules (black arrowhead) (scale bar, 50 μm). **j** Magnified image of the boxed area in **i** showing necrotic tubules (black arrowhead) (scale bar, 30 μm). **k** Kidney section of AFSC-treated rats on day 12 after cisplatin injection showing almost normal architecture of the tubules and preservation of the integrity of the cellular structure (scale bar, 50 μm). **l** Jablonski grading score for the assessment of renal tubular cell necrosis in saline- and AFSC-treated kidneys on day 8 and day 12 after cisplatin injection. Values expressed as mean ± SE (*p < 0.05 versus saline-treated group)

On the 5th day of cisplatin injection, rats exhibited a significant increase in the BUN and serum creatinine levels as compared to healthy controls (*p* < 0.05). On day 8, a significant decrease in the BUN and serum creatinine levels was observed in the AFSC-treated group as compared to the saline-treated group (*p* < 0.05), but both groups had significantly higher levels as compared to healthy controls (*p* < 0.05). On day 12, the blood biochemical parameters in the AFSC-treated group became comparable to that of healthy controls, but they were still significantly higher in the saline-treated group as compared to healthy controls (*p* < 0.05) (Fig. 2a, b). Histopathological analysis of kidney tissues revealed that, on the 5th day of cisplatin injection, the kidneys of ARF rats exhibited severe tubular necrosis with the loss of brush border, hyaline cast formation, and tubular dilatation (Fig. 2d, e). On day 8, the kidneys exhibited significant attenuation of tubular injury in the AFSC-treated group as compared to the saline-treated group (Fig. 2f, h). The Jablonski’s histological score also revealed significantly lower necrosis in the kidneys of AFSC-treated rats as compared to saline-treated rats (*p* < 0.05) (Fig. 2l). On day 12, the kidneys of saline-treated rats still showed necrotic tubular cells and hyaline casts, but the kidneys of AFSC-treated rats showed a lower index of cellular damage indicating attenuation of renal injury by infused cells (Fig. 2i, k) and there was a significant difference in the Jablonski grading score between the AFSC-treated group and saline-treated group (*p* < 0.05) (Fig. 2l).

The homing capacity of AFSC to the injured kidney was evaluated by the presence of GFP-labeled cells in the renal parenchyma 3 days after their administration. Their frequency averaged 2.2 ± 0.77 per 10^5^ renal cells. AFSC were predominantly found to localize in the peritubular areas and rarely within the tubular epithelium (Additional file 1: Figure S2).

### Administration of AFSC reduces apoptosis in kidney tissues

**Fig. 3:**
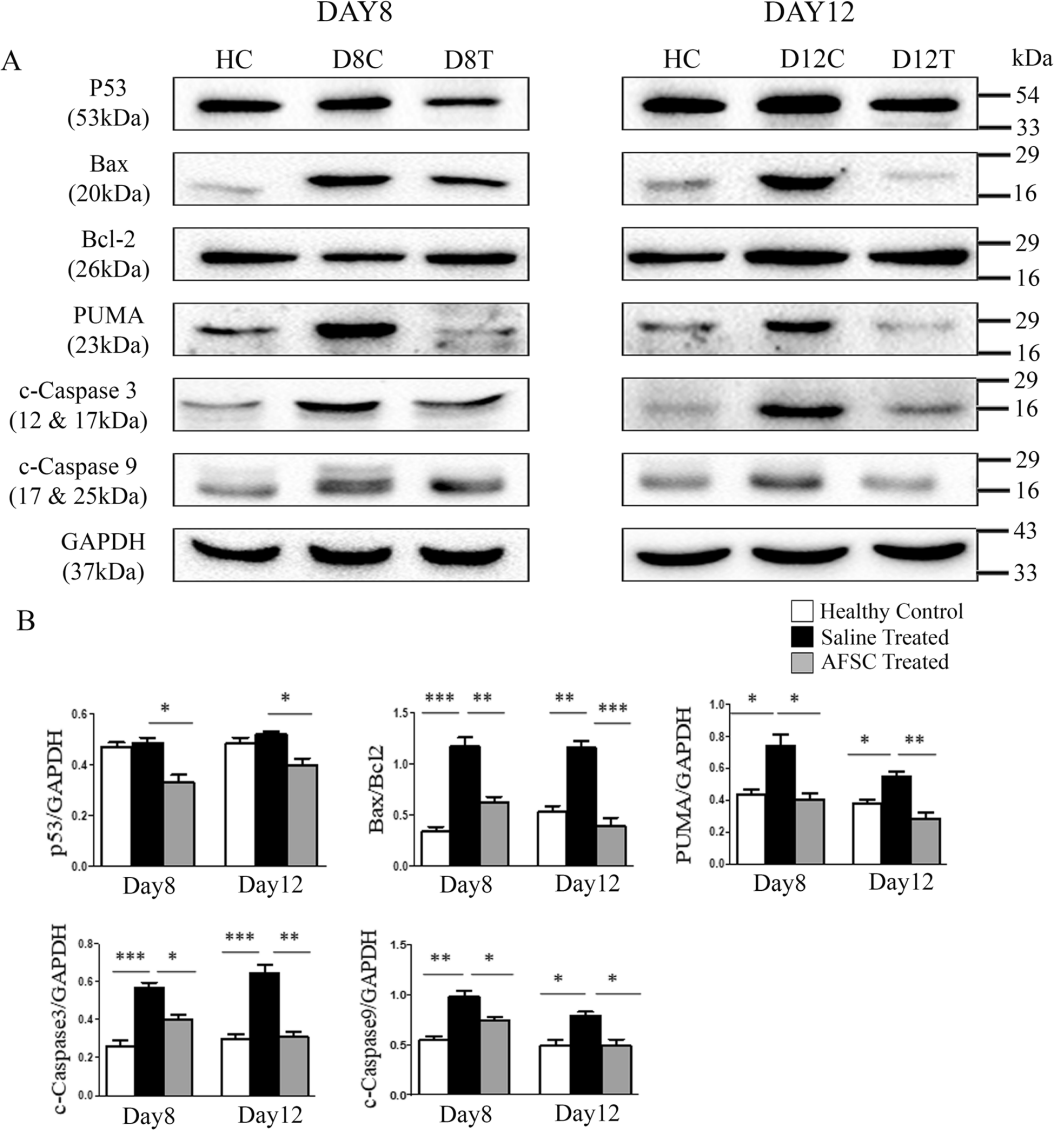
AFSC inhibit apoptosis in kidneys with ARF. **a** Representative immunoblots showing the expression of apoptotic proteins, viz, p53, Bax, Bcl-2, PUMA, cleaved caspase-3, and cleaved caspase-9, in kidney tissues of healthy control, saline-treated, and AFSC-treated groups on day 8 and day 12 after cisplatin injection. **b** Bar diagrams showing semi-quantitative densitometric determination of ratio of expression of Bax/Bcl-2 and expression of apoptotic proteins, viz, p53, PUMA, cleaved caspase-3, and cleaved caspase-9, by referring each gene to GAPDH which was taken as internal control. Values expressed as mean ± SE of three independent blots (*p < 0.05, **p < 0.01, ***p < 0.001)

Western blot analysis was performed to determine the levels of apoptosis-related signaling pathway proteins on day 8 and day 12 after cisplatin injection. A significant upregulation in the expression levels of pro-apoptotic proteins, viz, PUMA (*p* < 0.05 for both day 8 and day 12), Bax/Bcl2 ratio (*p* < 0.001 for day 8; *p* < 0.01 for day 12), cleaved caspase-3 (*p* < 0.001 for both day 8 and day 12), and cleaved caspase-9 (*p* < 0.01 for day 8; *p* < 0.05 for day 12), was observed in the saline-treated group as compared to healthy controls. However, after administration of AFSC, there was a marked downregulation of all the pro-apoptotic proteins in the AFSC-treated group as compared to the saline-treated group (p53: *p* < 0.05 for both day 8 and day 12; PUMA: *p* < 0.05 for day 8; *p* < 0.01 for day 12; Bax/Bcl2 ratio: *p* < 0.01 for day 8; *p* < 0.001 for day 12; cleaved caspase-3: *p* < 0.05 for day 8; *p* < 0.01 for day 12; cleaved caspase-9: *p* < 0.05 for both day 8 and day 12) (Fig. 3).

**Fig. 4:**
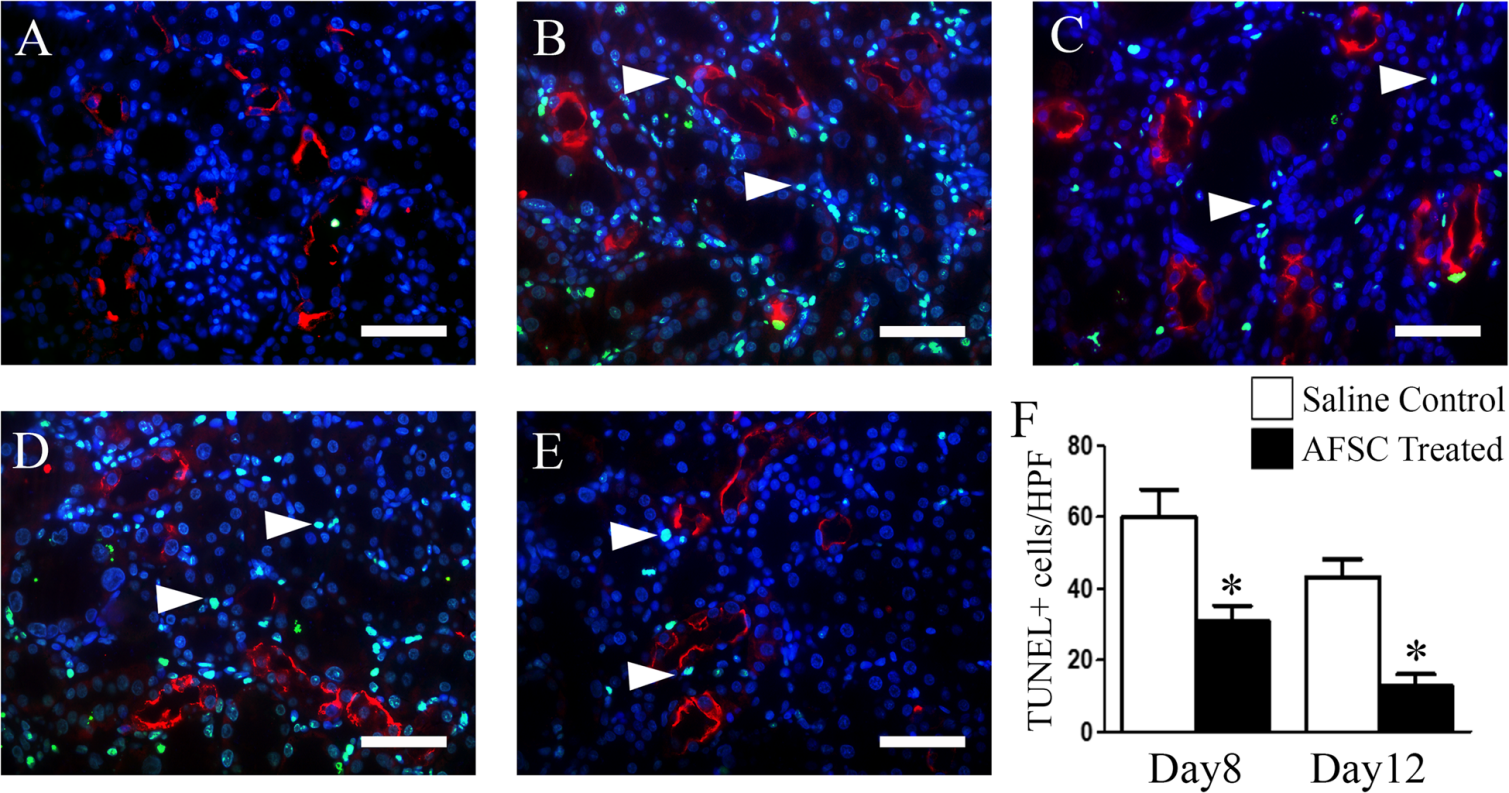
Effect of AFSC therapy on apoptosis of renal tubular epithelial cells in cisplatin-injured kidney. Representative immunofluorescence photomicrographs showing TUNEL-positive cells (green, indicated by white arrowheads) in the cortical region of the kidney tissue of **a** healthy control, **b** saline-treated group (day 8), **c** AFSC-treated group (day 8), **d** saline-treated group (day12), and **e** AFSC-treated group (day 12). The sections were co-stained with LTL (red) to mark the proximal tubule and Hoechst for nuclei (blue) (scale bar, 30 μm). **f** Quantification of TUNEL-positive cells in kidney sections of saline-treated and AFSC-treated groups on day 8 and day 12 after cisplatin injection. Values expressed as mean ± SE (*p < 0.01)

Corroborating with our western blot data, TUNEL-positive cells in the kidneys of AFSC-treated rats were significantly lower as compared to that of saline-treated rats on day 8 (*p* < 0.01), which further decreased significantly (*p* < 0.01) on day 12 (Fig. 4).

### AFSC mediate activation of autophagy in cisplatin-induced ARF rats

**Fig. 5:**
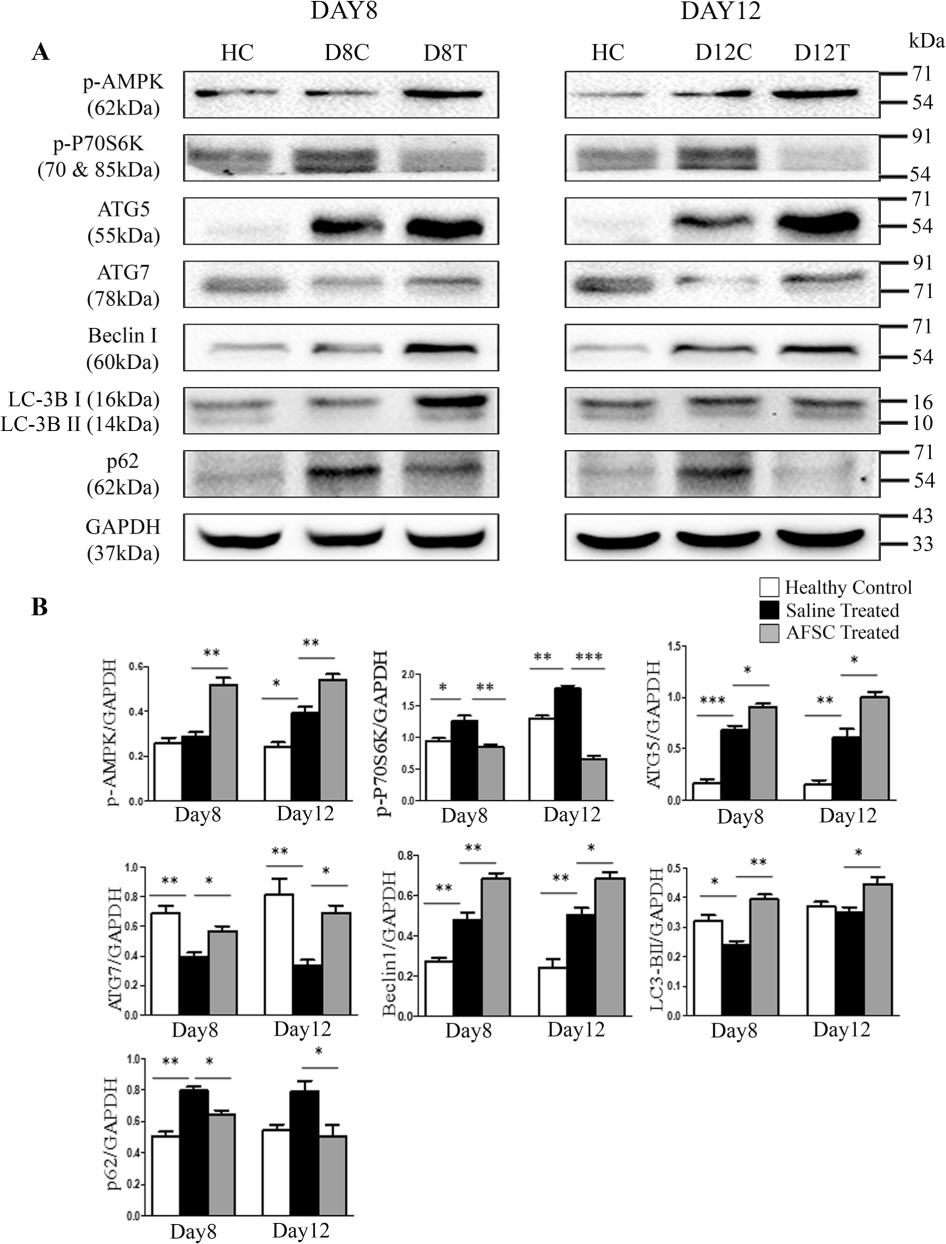
AFSC mediate activation of autophagy in response to cisplatin-induced acute renal failure. **a** Representative immunoblots showing the expression of the autophagic proteins, viz, phospho-AMPK, phospho-p70S6K, ATG5, ATG7, Beclin1, LC3B, and p62, in kidney tissues of healthy control, saline-treated, and AFSC-treated groups on day 8 and day 12 after cisplatin injection. **b** Bar diagrams showing semi-quantitative densitometric analysis for comparison of autophagic protein expression by referring each gene to GAPDH which was taken as internal control. Values expressed as mean ± SE of three independent blots (*p < 0.05, **p < 0.01, ***p < 0.001)

Since induction of autophagy rescues from cisplatin-induced injury [12], we next studied if AFSC-mediated protection involved autophagy induction. AMPK and mammalian target of rapamycin (mTOR) are the critical regulators of autophagy [17]. We demonstrated the effect of AFSC therapy on the levels of phospho-AMPK and on the downstream target of mTOR signaling pathway, i.e., phospho-p70S6K. The levels of phospho-AMPK were found to be significantly upregulated in the AFSC-treated group as compared to the saline-treated group (*p* < 0.01), while the levels of phospho-p70S6K were found to be significantly reduced after AFSC administration as compared to the saline-treated group (*p* < 0.01 for day 8; *p* < 0.001 for day 12). These results indicate that AFSC therapy inhibits mTOR downstream target, i.e., phospho-p70S6K, and activates phospho-AMPK, thereby activating autophagy in the renal tubular epithelial cells in response to cisplatin. To determine the level of autophagy activation following AFSC therapy, we examined the expression of autophagy-related proteins, viz, ATG5 and ATG7, and other critical autophagy markers including Beclin-1, LC3-II, and p62 on both day 8 and day 12. The levels of autophagy-related proteins, i.e., ATG5 and ATG7, were found to be significantly increased in the AFSC-treated group as compared to the saline-treated group (*p* < 0.05). The levels of autophagy markers, viz, LC3B-II and Beclin-1, were also found to be significantly increased in the AFSC-treated as compared to the saline-treated group (*p* < 0.01 for day 8; *p* < 0.05 for day 12) while the levels of p62 were found to be significantly decreased in the AFSC-treated group as compared to the saline-treated group (*p* < 0.05) (Fig. 5).

In addition, the immunofluorescence staining for p62 and cleaved-caspase 3 in kidney tissue sections also revealed that there was decreased accumulation of p62 substrate along with decreased expression of cleaved-caspase3 in the AFSC-treated group as compared to the saline-treated group on both day 8 and day 12, which corroborated with our western blot results (Additional file 1: Figure S3).

### Amniotic fluid stem cells prevent cisplatin-induced apoptosis by activating autophagy in-vivo

**Fig. 6:**
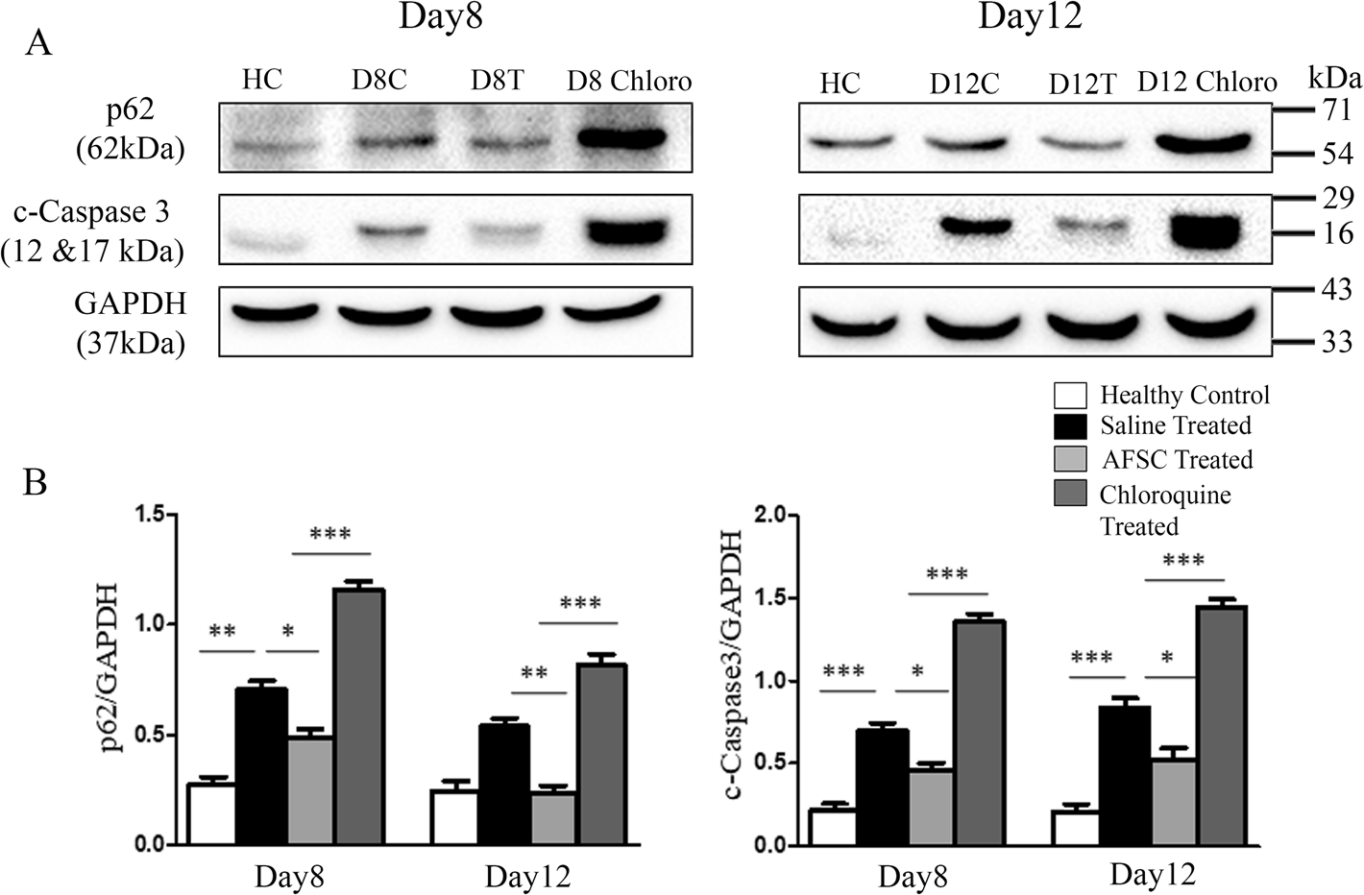
Effect of chloroquine administration on autophagy and apoptosis. **a** Representative immunoblots showing the expression of autophagic marker p62 and apoptosis marker cleaved caspase-3 in kidney tissues of healthy control, saline-treated, AFSC-treated, and chloroquine-treated groups on day 8 and day 12 after cisplatin injection. **b** Bar diagrams showing semi-quantitative densitometric analysis for comparison of expression of p62 and cleaved caspase-3 by referring each gene to GAPDH which was taken as internal control. Values expressed as mean ± SE of three independent blots (*p < 0.05, **p < 0.01, ***p < 0.001)

In order to confirm whether the preventive effects of AFSC on cisplatin-induced apoptosis are dependent on autophagy, we used an autophagic inhibitor, i.e., chloroquine, and analyzed its effect on the autophagic flux marker: p62, and the apoptosis marker: cleaved caspase-3. The protein levels of the autophagic substrate p62 and the apoptotic marker cleaved caspase-3 were found to be significantly upregulated in the chloroquine-treated group as compared to the therapy-treated group on both day 8 and day 12 (*p* < 0.001), indicating that inhibition of autophagy by chloroquine (as evident by p62 accumulation) leads to enhanced renal apoptosis (as evident by upregulated expressions of cleaved caspase-3) (Fig. 6).

**Fig. 7:**
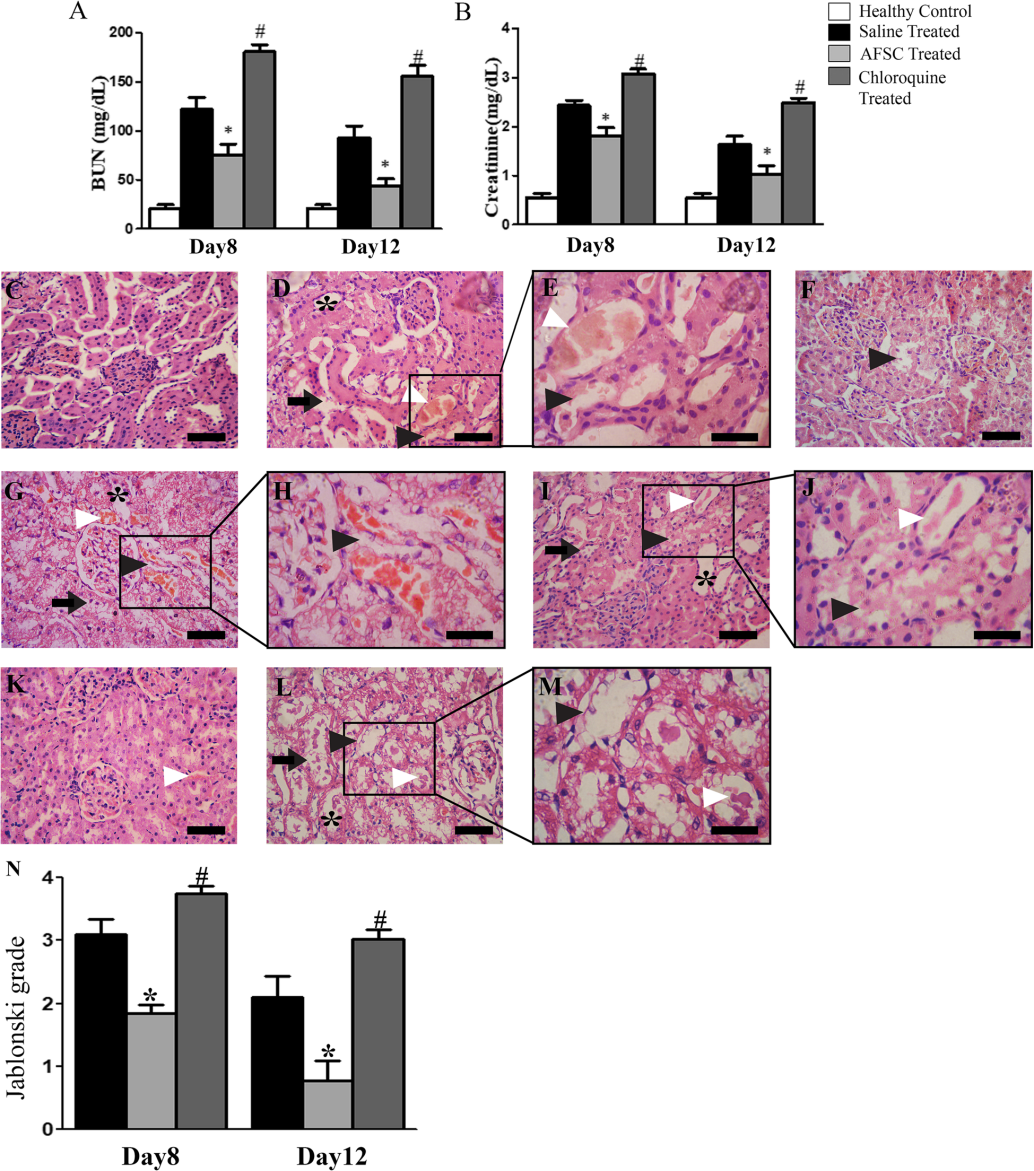
Effect of chloroquine administration on renal function and histology in rats with cisplatin-induced ARF. **a** Levels of blood urea nitrogen (BUN) and **b** serum creatinine measured in healthy controls, saline-treated, AFSC-treated, and chloroquine-treated ARF rats on days 8 and 12 following cisplatin injection. Values expressed as mean ± SE (*p < 0.05 versus saline-treated group; ^#^*p* < 0.001 versus therapy-treated group). Representative photomicrographs showing histology of cortical kidney tissue sections from healthy control (**c**), day 8 saline-treated ARF group (**d**) showing necrotic tubules (black arrowhead) and intratubular hyaline casts (white arrowhead) in higher magnification (**e**), day 8 AFSC treated ARF group (**f**), day 8 chloroquine-treated ARF group (**g**) showing necrotic tubules (black arrowhead) in higher magnification (**h**), day 12 saline-treated ARF group (**i**) showing necrotic tubules (black arrowhead) and intratubular hyaline casts (white arrowhead) in higher magnification (**j**), day 12 AFSC-treated ARF group (**k**), and day 12 chloroquine-treated ARF group (**l**) showing necrotic tubules (black arrowhead) and intratubular hyaline casts (white arrowhead) in higher magnification (**m**). The kidney sections of day 8 and day 12 chloroquine-treated ARF rats show severe tubular necrosis (black arrowhead) with intratubular hyaline casts (white arrowhead), tubular dilatation (asterisk), and loss of brush border (black arrow) as compared to other groups. **n** Jablonski grading score for the assessment of tubular necrosis in saline-treated, AFSC-treated, and chloroquine-treated groups on day 8 and day 12 after cisplatin injection. Values expressed as mean ± SE (*p < 0.05 versus saline-treated group; ^#^*p* < 0.001 versus AFSC-treated group)

We also analyzed the effect of chloroquine on renal function and histology on both day 8 and day 12 after cisplatin injection. Administration of chloroquine induced a severe loss of renal function, which was observed by a significant increase in the BUN and serum creatinine levels in the chloroquine group as compared to the therapy-treated group (*p* < 0.001) on both day 8 and day 12 (Fig. 7a, b). Consistent with our functional data, the histological examination also showed that the tubular damage following cisplatin-induced renal injury was further aggravated by chloroquine treatment and more tubules showed dilatation and distortion, loss of brush border, hyaline casts, and sloughed debris in the lumen space as compared to other groups (Fig. 7d–m). Furthermore, there was a significant difference in the Jablonski grading score between the chloroquine-treated group and therapy-treated group (*p* < 0.001) on both day 8 and day 12 (Fig. 7n). Collectively, these results link the induction of autophagy by AFSC with their beneficial effects in cisplatin-induced renal injury.

## Discussion

The present study demonstrated that culture-expanded rat AFSC express mesenchymal and renal progenitor markers. The administration of AFSC in cisplatin-induced rat model of ARF resulted in improvement of renal function and attenuation of renal damage. The infused AFSC activated autophagy, which led to reduction in renal cell apoptosis and acceleration in renal recovery. The protective effects of AFSC were blunted by the use of an autophagic inhibitor, i.e., chloroquine, suggesting that induction of autophagy is essential for the protective role of AFSC in cisplatin-induced renal injury. To the best of our knowledge, this is the first study which demonstrates activation of autophagy as one of the important mechanisms responsible for the renoprotective effects of AFSC in cisplatin-induced AKI.

In our recent study, we characterized rat AFSC for the expression of renal progenitor markers, viz, WT1, PAX2, SIX2, CITED1, and SALL1, and evaluated their in vitro potential to differentiate into renal proximal tubular epithelial-like cells and juxtaglomerular-like cells [5]. In the present study, we have further evaluated the therapeutic potential of AFSC in alleviating cisplatin-induced ARF and the underlying mechanisms for the renoprotective effects. However, previous studies have reported the renoprotective effects of AFSC in different pre-clinical models of renal diseases [9, 11, 18], but their mechanism of action still remains unclear. AFSC have been shown to exert their renoprotective effects by their ability of homing to the injured kidney and paracrine release of various soluble factors that create a regenerative microenvironment [7, 19] or through their ability to transdifferentiate into cells expressing proximal and distal tubular agglutinins [11]. We also performed tracking experiments to determine the homing ability of the administered AFSC and found that GFP-positive cells predominantly localized in the peritubular areas of the injured kidney, which may represent only a small fraction of the infused cells, as a large fraction of the intravenously infused cells, get trapped into the lungs, liver, and spleen as reported for other cell types [20–22].

Recently, our group showed the renoprotective effect of fetal kidney stem cells in ischemia and cisplatin-induced rat models of ARF via their anti-inflammatory, anti-apoptotic, anti-oxidative, and angiogenic properties [23, 24]. The present study also highlights that AFSC promote amelioration of cisplatin-induced kidney injury via their anti-apoptotic properties. Apoptosis of tubular cells is the characteristic feature of cisplatin nephrotoxicity and has been observed both in vitro and in vivo [25, 26]. Several therapeutic interventions targeting the apoptotic pathways involved in AKI have demonstrated the beneficial effects on both in vitro cultured renal tubular cells [27] and in vivo animal models of cisplatin-induced renal injury [21, 28]. In the present study, we demonstrated that the number of TUNEL-positive cells increased significantly after cisplatin administration. However, infusion of AFSC resulted in a significant reduction of these TUNEL-positive cells. The decreased expression of cleaved caspase-3 and cleaved caspase-9 also confirmed that infusion of AFSC reduced apoptosis in the kidneys injured by cisplatin. Cisplatin administration also activates p53 which in turn promotes apoptosis by upregulating the expression of PUMA, one of the major downstream mediators for the apoptotic actions of p53 [29]. Knockout studies have shown suppression of cisplatin-induced apoptosis in PUMA knockout cells, indicating that inhibition of PUMA inhibits apoptosis [30]. We observed that administration of AFSC attenuated the activation of p53 and its downstream target PUMA, suggesting the protective effect of AFSC in cisplatin-induced AKI. Cisplatin administration activates Bax, reduces Bcl2, and shifts the Bax/Bcl2 ratio in a pro-apoptotic direction. Bax deletion has been shown to confer resistance to cisplatin in animals, further highlighting the pathological role of Bax in cisplatin nephrotoxicity [31]. Our study demonstrated that administration of AFSC upregulated the anti-apoptotic protein, Bcl2, and downregulated the pro-apoptotic protein Bax, which corresponds with reduced apoptosis and improved renal function.

Under basal or physiological conditions, autophagy works as a cellular housekeeper and helps in eliminating damaged organelles and intracellular pathogens and contributes to the maintenance of cellular homeostasis and quality control of proteins and sub-cellular organelles. However, under pathological conditions or cell stress, autophagy is induced and serves as a protective mechanism for cell survival [32]. The role of autophagy in the pathogenesis of AKI still remains controversial, as there are contradictory reports regarding the same [33]. Some reports have suggested a cytoprotective role of autophagy during cisplatin treatment [34] while the others have shown that autophagy may be involved in apoptosis of the proximal tubular cells following cisplatin treatment [35]. However, our results establish a renoprotective role of autophagy in cisplatin-induced kidney injury. Our observation is in concordance with previous studies that have reported that autophagy triggers a pro-survival response in cisplatin-induced AKI [12, 34]. However, whether AFSC can activate autophagy to prevent renal tissue injury has not been explored. Here, we provide evidence for the first time that AFSC activate autophagy in response to cisplatin-induced AKI. The autophagic response to cisplatin was identified by monitoring the autophagic flux which was determined by the LC3-II and p62 expression levels. The AFSC therapy significantly increased LC3-II expression level and decreased the expression level of p62. The LC3-II levels directly correlate with the autophagosome number while the levels of p62 inversely correlates with the autophagic activity [36]. The stem cell therapy also significantly increased the expression of other autophagy-related proteins, i.e., ATG5, ATG7, and Beclin-1. ATG5 and ATG7 proteins form the critical component of the autophagic pathway that are involved in the elongation and closure of the autophagosomal membrane [37]. Beclin-1 is the mammalian homolog of ATG6 and is involved in the vesicle nucleation, an early event during autophagosome formation [38]. Previous studies have shown that knockdown of the autophagy proteins: Beclin-1 and ATG5, leads to enhanced activation of caspases and tubular cell apoptosis during cisplatin treatment [39]. The expression levels of phospho-AMPK were found to be significantly elevated, while that of phospho-p70S6K were found to be significantly downregulated by AFSC therapy treatment, suggesting that AFSC may activate autophagy via activation of AMPK pathway and inhibition of the mTOR signaling pathway. Previous studies have also shown that cisplatin-induced tubular cell apoptosis can be effectively ameliorated by AMPK activation and inhibition of mTOR signaling pathway [40–42]. A recent study by Wang et al. showed that human umbilical cord mesenchymal stem cell-derived exosomes (hucMSC-Ex) prevent cisplatin-induced apoptosis under both in vitro and in vivo conditions through the activation of autophagy via inhibition of the mTOR signaling pathway and its downstream target, i.e., p70S6K [43]. In the same line, Jia et al. also reported that hucMSC-Ex prevents cisplatin-induced renal injury through activation of autophagy via. trophic factor 14-3-3ζ which interacts with ATG-16 L [44], indicating that stem cell secretomes have therapeutic effects on renal injury via autophagy activation. These studies suggest that paracrine mediators of renal cell autophagy may include components of AFSC’s secretome such as miRNA and other soluble proteins that link the efficacy of AFSC therapy with activation of autophagy.

To further investigate whether autophagy was involved in the protective effects of AFSC against cisplatin-induced apoptosis, chloroquine, a pharmacological inhibitor of autophagy, was administered. Chloroquine, a widely used anti-malarial drug, is known to block the last phase of autophagy by inhibiting the autophagosome fusion with lysosome and thus slows down the lysosomal acidification [45]. In the present study, we observed that chloroquine administration inhibited the protective effects of AFSC therapy and led to a further worsening in the renal structure and function caused by cisplatin by significantly increasing the expression levels of p62 and cleaved caspase-3, thereby suggesting the renoprotective role of autophagy in this disease model. Our observation corroborates with a previous study which showed that administration of chloroquine abolished the protective effects of neferine against cisplatin-induced apoptosis [46]. However, a recent study by Mauthe et al. (2018) showed that hydroxychloroquine at a dose of 60 mg/kg in mice leads to multiple structural alterations like golgi disorganization in the kidney and intestinal cells in an autophagy-independent manner [47]. This may hold true for the present study as well, that effects of chloroquine may not be only limited to inhibition of autophagy, but autophagy-independent effects of chloroquine may also be responsible that may contribute to worsening of renal injury after chloroquine application. Therefore, further studies are needed in future to determine other ultrastructural alterations that may be caused by chloroquine application for blocking autophagy and their functional consequences. Likewise, other pharmacological inhibitors of autophagy such as 3-methyladenine (3-MA) have been equally shown to exhibit off-target effects [48]; therefore, alternative ways for blocking autophagy, like the use of autophagy gene-knockout animal models, would be more suitable to determine the actual involvement of autophagy in amelioration of cisplatin-induced acute renal failure. However, this was beyond the scope of the present study.

## Conclusions

In conclusion, the present study demonstrates that administration of AFSC in cisplatin-induced ARF results in rapid recovery of renal function and histology by activation of autophagy and inhibition of apoptosis. To the best of our knowledge, this is the first study that highlights autophagy as one of the important mechanisms by which AFSC induce a renoprotective effect in cisplatin-induced ARF. However, further studies on the long-term effects of AFSC in different pre-clinical models of ARF would be important for its translational implications into clinical settings for the development of novel pharmacological therapies for ARF. Moreover, alternative ways for blocking autophagy, like use of autophagy gene-knockout animal models, would be more suitable to determine the conclusive evidence for the involvement of autophagy in amelioration of cisplatin-induced kidney injury.

## Supplementary information

**Additional file 1: Figure S1.** Experimental Schedule. **Figure S2.** In-vivo tracking of GFP-labelled AFSC in cisplatin-injured kidney tissue. **Figure S3.** AFSC therapy mediates activation of autophagy and inhibition of apoptosis in response to cisplatin-induced renal injury.

## Data Availability

All relevant data related to this study has been included in the article.

## Abbreviations

AFSC: Amniotic fluid stem cells
ARF: Acute renal failure
AKI: Acute kidney injury
CKD: Chronic kidney disease
MSC: Mesenchymal stem cells
MEM: Minimum essential media
FITC: Fluorescein isothiocyanate
PE: Phycoerythrin
BUN: Blood urea nitrogen
H&E: Hematoxylin and eosin
SDS-PAGE: Sodium dodecyl sulphate-polyacrylamide gel electrophoresis
HRP: Horseradish peroxidase
TUNEL: Terminal deoxynucleotidyl-transferase-mediated dUTP nick end labeling
HPF: High power field
mTOR: Mammalian target of rapamycin
hucMSC-Ex: Human umbilical cord mesenchymal stem cell-derived exosomes
SE: Standard error
ANOVA: One-way analysis of variance
LTL: *Lotus tetragonolobus* Lectin
3-MA: 3-Methyladenine
