# Supplementary material for: Amniotic fluid stem cells ameliorate cisplatin-induced acute renal failure through induction of autophagy and inhibition of apoptosis
Source: Stem Cell Res Ther. 2019 Dec 4;10:370. doi: 10.1186/s13287-019-1476-6 (PMC6894207; doi:10.1186/s13287-019-1476-6)
Supplement: Supplementary file 1 — Additional file 1: Figure S1. Experimental Schedule. Figure S2. In-vivo tracking of GFP-labelled AFSC in cisplatin-injured kidney tissue. Figure S3. AFSC therapy mediates activation of autophagy and inhibition of apoptosis in response to cisplatin-induced renal injury. [file 13287_2019_1476_MOESM1_ESM.docx]

**Additional file**

**
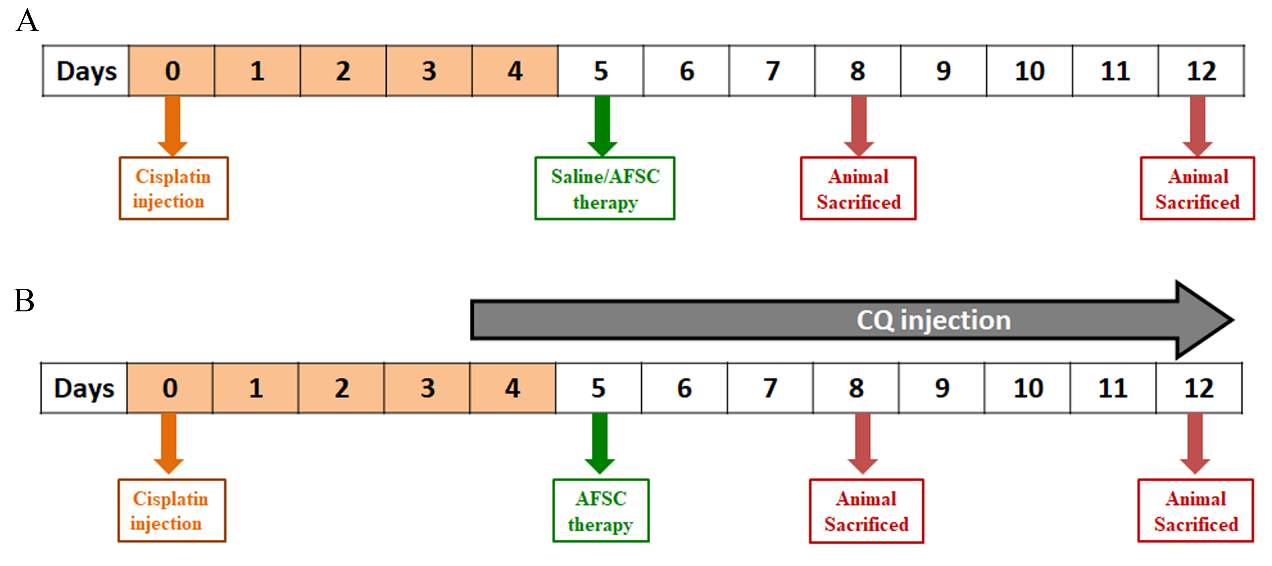
**

**Figure 1 (S1):**

**A:** Schematic representation of the protocol for cisplatin-induced acute kidney injury and treatment with amniotic fluid stem cells (AFSC). Cisplatin (7 mg/kg) was administered intraperitoneally at Day 0. The arrow at Day 5 indicates the time point for administration of AFSC (2x10^6^ cells/rat) or vehicle alone and the subsequent arrows indicate the time of sacrifice after 8 days and 12 days of cisplatin injection; **B:** Schematic representation of the protocol for chloroquine administration to confirm the protective effects of autophagy following cisplatin induced AKI. Chloroquine (60mg/kg) was administered intra-peritoneally to rats one day prior therapy and then daily till sacrifice. The arrows indicate the time points for cisplatin administration, AFSC administration and sacrifice.

**
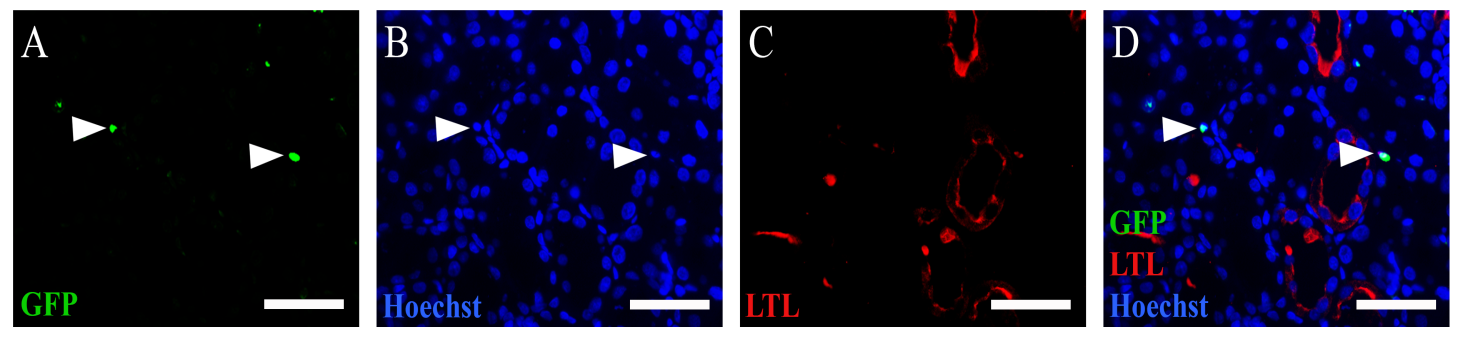
**

**Figure 2 (S2):**

Representative photomicrograph (Scale bar: 30µm) showing the localization of GFP-labeled AFSC (green; white arrow head) in cisplatin-injured kidney tissue. The sections were co-stained with LTL to mark the proximal tubules and Hoechst for nuclei. The four panels from left to right show GFP as green, Hoechst/nuclei as blue, LTL as red and the merged image, respectively.


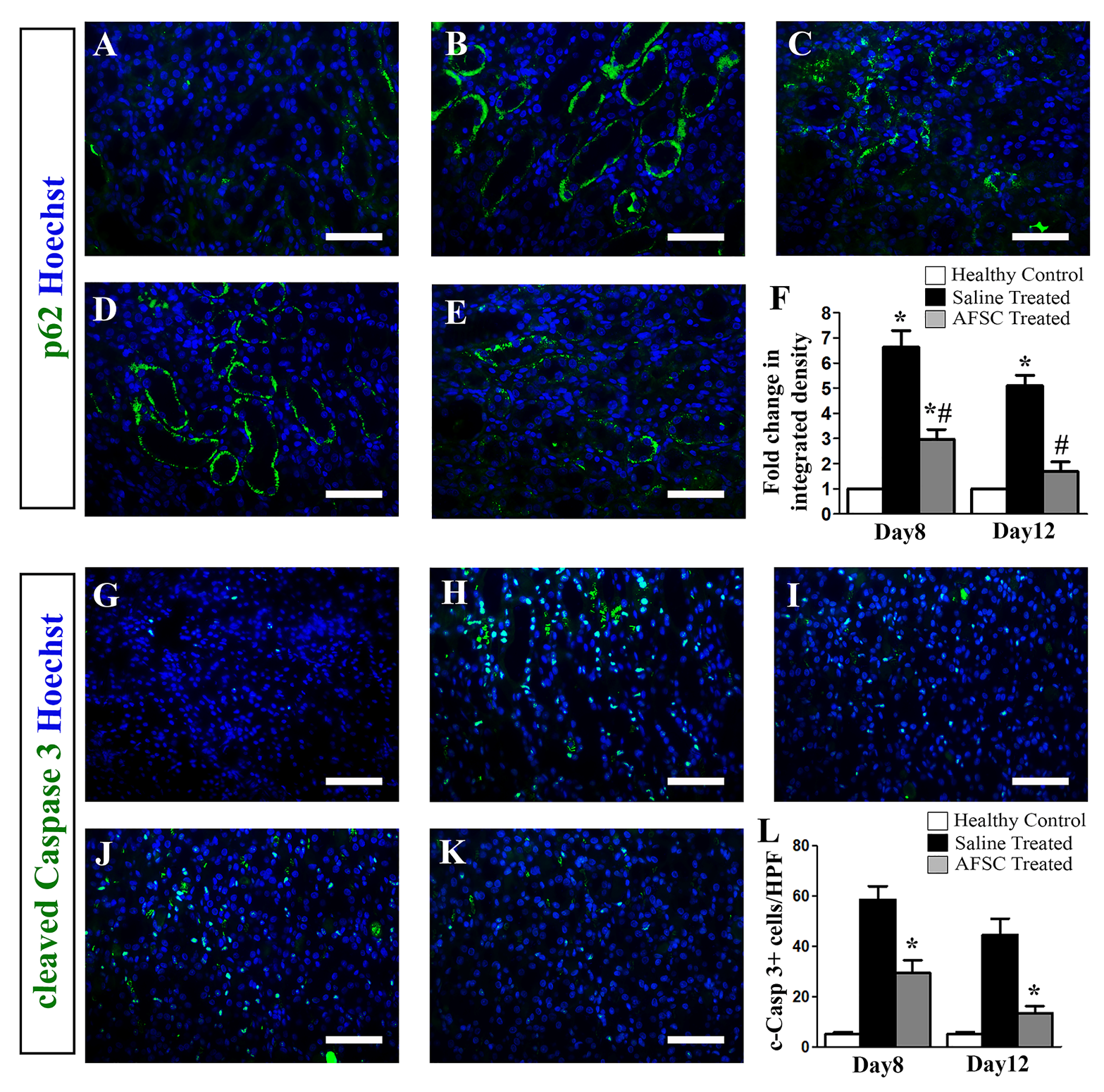


**Figure 3 (S3):**

Representative immunoflorescence photomicrographs of p62 staining of kidney tissue sections from **A:** healthy control; **B:** saline treated group (Day 8); **C:** AFSC treated groups (Day 8); **D:** saline treated group (Day12); **E:** AFSC treated group (Day 12); **F:** Quantification of p62 staining in kidney sections of saline treated and AFSC treated groups on Day 8 and Day 12 after cisplatin injection. Values expressed as Mean ± SE (*p<0.001 vs. healthy controls; #p<0.001 vs. saline treated group). Representative immunoflorescence photomicrographs (Scale bar: 30µm) of cleaved-caspase3 staining of kidney sections from **G:** healthy control; **H:** saline treated group (Day 8); **I:** AFSC treated groups (Day 8); **J:** saline treated group (Day12); **K:** AFSC treated group (Day 12); **L:** Quantification of cleaved-caspase 3 positive cells in kidney sections of saline treated and AFSC treated groups on Day 8 and Day 12 after cisplatin injection. Values expressed as Mean ± SE (*p<0.001 vs. saline treated group).
